# Psychological aspects of atrial fibrillation: A systematic narrative review: Impact on incidence, cognition, prognosis, and symptom perception

**DOI:** 10.1007/s11886-020-01396-w

**Published:** 2020-09-10

**Authors:** Karl-Heinz Ladwig, Andreas Goette, Seryan Atasoy, Hamimatunnisa Johar

**Affiliations:** 1 Department of Psychosomatic Medicine and Psychotherapy, Klinikum rechts der Isar, Technische Universität München (TUM), Langerstr. 3, 81675 Munich, Germany; 2 Institute of Epidemiology, Mental Health Research Unit, German Research Center for Environmental Health, Helmholtz Zentrum München, Neuherberg, Germany; 3 German Centre for Cardiovascular Research (DZHK), Partner site Munich Heart Alliance, Munich, Germany; 4 St. Vincenz-Krankenhaus GmbH, Medizinischen Klinik II, Paderborn, Germany; 5 Working Group on Molecular Electrophysiology, University Hospital Magdeburg, Magdeburg, Germany; 6 Department of Psychosomatic Medicine and Psychotherapy, University of Gießen and Marburg, Marburg, Germany

**Keywords:** Atrial fibrillation, Psychosocial stress, Cognition, Dementia, Symptom perception, Depression, Anxiety, PTSD, Autonomic regulation

## Abstract

**Purpose of the review:**

Atrial fibrillation (AF) is the most frequent arrhythmia in the general population. This review aims to provide a comprehensive overview of the psychological aspects of AF, compiling evidence from epidemiological, clinical, and basic research sources.

**Recent findings:**

Findings from large-scale population-based and clinical longitudinal studies reveal an association between negative affectivity (e.g. depression) and the incidence and clinical prognosis of AF. Studies investigating the impact of work stress parameters on AF onset show conflicting results. Researchers have reported the impact of AF on cognitive decline and on health-related quality of life, and have highlighted the role of interoceptive cues in the development of AF symptom burden and gender differences in psychological covariates of AF. Among biological pathways linking psychosocial factors to AF, research on autonomic regulation has yielded the most evidence so far, showing that the onset of AF is associated with simultaneous sympatho-vagal activation rather than an increase in vagal or sympathetic drive alone. Thus, modulation of the autonomic nervous system is likely to be a promising strategy for protecting the myocardium from pro-arrhythmic autonomic influences.

**Summary:**

In total, the findings show that AF is embedded as a disease condition in a psycho-societal context and is not an isolated medical problem per se. A broader perspective than a focus on the electrophysiology alone is urgently needed.

## 1.Introduction

Atrial fibrillation (AF) is defined as an uncoordinated atrial activation with consequent ineffective contractions. AF is an umbrella term for different forms classified in terms of duration and patterns of termination. AF episodes terminated within 7 days of onset are defined as paroxysmal, whereas AF episodes lasting longer are considered persistent, and those sustained for over 12 months as “long-standing persistent” AF.

AF is the most prevalent form of dysrhythmia, affecting an estimated >33 million people worldwide, with a global incidence of almost 5 million new AF cases each year [1]. A further increase is anticipated in the coming decades. For example, cases in the United States are expected to exceed 12 million by 2030 [2], and a recent investigation in the European Union (EU) [3] found an extraordinary nonlinear increase in both incidence and subsequent related health costs (mainly due to hospitalization expenditures). The number of AF cases is increasing with an aging population; an estimated 51.2% of cases in the EU in 2016 were in persons aged 80 years and older [3]. Notably, the true prevalence is likely even greater, due to a high proportion of “silent” and thus undetected cases (see subchapter 3.2.).

An emerging body of literature suggests a close interaction between psychological factors and AF disease condition, which is likely to be of meaningful importance for understanding and treating AF patients. The present review aims to assemble this evidence systematically, including the effects of psychological factors on incident AF and their prognostic role in the long-term course of the disease. The involvement of psychological factors in AF patients’ quality of life (QoL), independent of the objective parameters of the disease process and the impact of AF on cognitive decline and dementia, will be highlighted. Given the critical importance of AF symptom burden in treatment decisions, we show that AF symptoms are almost indistinguishably interwoven within a complex perceptive processing strongly fueled by affective conditions. We investigate gender differences in psychological covariates of AF and the current understanding of physiological stress response mechanisms that are probably related to AF, drawing on evidence from animal and human laboratory work, epidemiological studies, and clinical trials. We present an overview of interventional studies that have attempted to modify psychological or social factors to improve outcomes in patients with AF.

## 2.Impact of adverse psychological factors on AF incidence

In contrast to the multitude of therapeutic strategies dealing with a manifested AF disease condition, clinician awareness of AF as “*an avoidable condition*” is still a nascent concept [4]. Notably, somatic risk factors are currently receiving more attention—among them hypertension [5] and established lifestyle factors including excessive alcohol consumption [6] and smoking [7]. In contrast, mental health-related risk factors are not yet widely acknowledged, although the evidence (regarding sample size, follow-up duration, and effect strength) is impressive. Tables 1 and 2 list the impacts of psychosocial stress and negative affectivity as demonstrated in large prospective population-based studies, including >160 000 participants in psychosocial stress studies and even many more in studies dealing with affective conditions. As outlined in Table 1, data from a large Swedish longitudinal cohort (SLOSH-Study) show that job strain is a highly significant predictor of AF (odds ratio [OR] 1.93) when exposure time is >10 years [8], consistent with a study demonstrating a robust effect of long working hours, a marker for stress-related work organization conditions, on incidence of AF. A large effect size for severe self-perceived stress (OR 1.60) is found in the REasons for Geographic and Racial Differences in Stroke Study, while other more global psychological distress measures are not significant. Table 2 focuses on negative affectivity. While anger and anxiety may act as immediate triggers of paroxysmal AF in vulnerable patients, they only gain borderline significance in long-term studies. In contrast, depressed mood and vital exhaustion, along with antidepressant use (although other studies with conflicting evidence), were associated with an increased risk of incident AF. More recently, PTSD has received attention and demonstrates a relatively small, yet significant, effect size in a large study in veterans. Nevertheless, the evidence remains conflicting, as a number of studies did not observe significant effects of psychosocial stress [12, 13] and depression or antidepressant use [12, 15] on the incidence of AF.

**Table 1:** Impact of adverse psychological factors on AF incidence—findings from population-based longitudinal studies: Psychosocial stress factors

Study	Study type	Psychosocial factors	Sample size	Increase in risk
Fransson et al., 2018 [8]	Prospective study of working adults	Job strain	10,121	HR 1.11 (95% CI (0.67–1.83) before 10.7-year exposure HR 1.93 (95% CI 1.10–3.36) after 10.7-year exposure
Kivimäki et al., 2017 [9]	Prospective multi-cohort study	Long working hours	85,494	HR 1.42 (95% CI 1.13 –1.80)
Svensson, et al., 2017 [10]	Prospective population-based study	Psychological stress (job strain and non-occupational stress)	8765 men & 13,543 women	HR 1.05 (95% CI 0.86–1.28) for men HR, 1.15 (95% CI 0.95–1.39) for women
O'Neal et al., 2015 [11]	Prospective national cohort study	Perceived stress	25,530	OR 1.12 (95% CI 0.98–1.27) for low stress OR 1.27 (95% CI 1.11–1.47) for moderate stress OR 1.60 (95% CI 1.39–1.84) for high stress
Whang et al., 2012 [12]	Randomized trial	Global psychological distress	30,746 [women)	HR 0.99 (95% CI 0.72–1.35) for censored CVD events HR 0.92 (95% CI 0.66–1.29) for non-censored CVD events
Garg et al., 2019 [13]	Prospective population-based study	Chronic stress	6644	HR 1.06 (95% CI 0.89–1.27)

**Table 2:** Impact of adverse psychological factors on AF incidence—findings from population-based longitudinal studies: Negative affectivity

Study	Study type	Psychosocial factor	Sample size	Increase in risk
Garg et al., 2019 [13]	Multicenter prospective population-based study	Depressive symptoms	6644	HR 1.34 (95% CI 1.04–1.74)
Whang et al., 2012 [12]	Randomized trial	Proxy measure for depression (MHI-5 and/or antidepressant use)	30,746 women	HR 0.99 (0.78–1.25)
Garg et al., 2019 [13]	Multicenter prospective population-based study	Antidepressant use	6644	HR 1.36 (95% CI, 1.04–1.77)
Garg et al., 2019 [14]	Prospective population-based study	Antidepressant use	11,445	HR 1.21 (95% CI 0.98–1.50)
Lapi et al., 2015 [15]	Case–control cohort study	Current antidepressant use	116,125	RR 0.98 (95% CI 0.86–1.12)
Lapi et al., 2015 [15]	Case–control cohort study	Recent antidepressant use	116,125	RR 1.02 (95% CI 0.86–1.30)
Garg et al., 2019 [13]	Prospective population-based study	Anxiety	6644	HR 0.97 (95% CI 0.79–1.20)
Eakere et al., 2005 [16]	Prospective population-based cohort study	Anxiety	3682	HR 1.10 (95% CI 0.95–1.27) for men HR 1.03 (95% CI 0.81–1.31) for women
Rosman et al., 2019 [17••]	Prospective veteran population study	PTSD	988,090	HR 1.13 (95% CI, 1.02–1.24)
Patton et al., 2011 [18]	Census mortality data	Early life factors (place of birth within the US stroke belt)	95.6 million	OR 1.19 (95% CI 1.13, 1.25) for African-American and 1.09 (CI 1.07, 1.12) for white population
Whang et al., 2012 [12]	Randomized trial post hoc analysis	Felt happy (Mental Health Inventory-5)	30,746 (women)	HR 0.69 (95% CI 0.49–0.99) for those who felt happy some or most of the time
Graff et al., 2016 [19]	Prospective population-based case control study	Partner bereavement	88,612	OR 1.90 (95% CI 1.34–2.69) for 8–14 days after loss OR 1.41 (95% CI 1.17 to 1.70) for 30 day after loss *results reduced but remained significant within 1 year after loss
Eaker et al., 2004 [20]	Prospective population-based cohort study	Trait anger, hostility, symptoms of anger	3873	HR 1.1 (95% CI: 1.0–1.4) for trait anger; HR 1.3 (95% CI: 1.1–1.5) for hostility; HR 1.2 (95% CI: 1.0–1.4) for symptoms of anger
Garg et al., 2020 [14]	Prospective population-based study	Anger	11,445	HR 1.08 (95% CI 0.92–1.27)
Garg et al., 2019 [13]	Prospective population-based study	Anger	6644	HR 0.95 (95% CI 0.77–1.17)
Eaker et al., 2005 [16]	Prospective population-based study	Tension	3682	Men 1.24 (95% CI 1.04–1.48) Women 0.83 (95% CI 0.63–1.11) 1
Garg et al., 2020 [14]	Prospective population-based study	Vital exhaustion	11,445	HR 1.20 (5% CI 1.06–1.35)
Garg et al., 2020 [14]	Prospective population-based study	Poor social ties	11,445	HR 1.09 (95% CI 0.94–1.27) for socially isolated individuals

## Psychosocial consequences of AF

### Cognition

Although asymptomatic in the vast majority of patients, AF can manifest as ischemic stroke, with up to fivefold increased stroke risk in the general population [21]. However, clinically diagnosed stroke represents only the tip of the iceberg, as AF-induced brain ischemia and silent brain infarcts [22] are associated with cognitive impairment and dementia [23] as well. Increasing evidence from meta-analyses that include large prospective epidemiological studies demonstrate that AF may be a predictor of cognitive impairment and increased risk of dementia (hazard ratios [HR] ranging from 1.3 to 2.3), with or without a history of stroke [24–29].

In a most recent meta-analysis of 16 studies including 2,415,356 individuals, subgroup analyses reveal that the association between AF and dementia risk appears to be stronger in studies with a follow-up duration > 5 years [29]. In the Rotterdam study, although the risk of all-cause dementia was highest for people who experienced the longest duration of AF, this dose–response relationship was only present in younger participants [30]. Similarly, data from the Whitehall II study confirm that patients with incident AF reach thresholds of cognitive impairment or dementia at an earlier age than patients with no history of AF, and individuals with longer exposure to AF (5–15 years) experience a faster cognitive decline [31]. Thus, the relationship to dementia is stronger when AF starts in middle age and when its duration is longer.

Beyond shared risk factors, AF may accelerate cognitive decline and increase the risk of dementia through multiple pathways. Apart from the major role of clinical stroke in AF-associated brain ischemia, the most likely mechanism is covert embolism to the brain [22, 32]. Moreover, AF has been associated with atrophy of brain volume [33] and altered hemostatic function favoring thrombosis [34].

Secondly, a role of cerebral hypoperfusion is also plausible. The beat-to-beat variations with reduced cardiac output present in AF rhythm may result in transient or chronic cerebral hypoperfusion [35, 36]. Cerebral hypoperfusion in AF may contribute to mechanistically exacerbated amyloid-beta and tau neuropathology, all contributing to cognitive impairment. Of note, the effect is more profound with persistent/permanent AF than paroxysmal AF, and with increasing AF burden [33]. Thirdly, systemic inflammation has been associated with hypercoagulation, endothelial dysfunction, and increased platelet activation, thus contributing to AF-related thromboembolism [37]. Finally, some evidence has emerged on the role of genetics, with single-nucleotide polymorphisms (SNPs) of genes associated with familial AF found to be related to the development of cognitive impairment [38].

Despite the role of oral anticoagulants as first-line medication in the prevention of ischemic stroke in patients with AF [39], it is still debatable whether the risk of AF-related dementia can be significantly reduced by oral anticoagulation treatment [40, 41]. Recent data suggest that the novel anticoagulants would be a better choice for the prevention of dementia than warfarin (a vitamin K antagonist) [42, 43], probably due to a lower rate of intracerebral bleeding [43]. Secondly, in terms of rhythm control strategies, cardioversion or AF ablation may result in sinus rhythm and improve cardiac output and cerebral perfusion. The Intermountain Study including 4212 patients who underwent catheter ablation for AF showed significantly lower incidence of Alzheimer’s dementia (0.2%) compared to a control group (0.9%) and non-AF patients (0.5%) (*p* < 0.001) [44]. A recent study also supports the protective role of rhythm control strategies in cognitive function [45], but strategies using antiarrhythmic drugs have inherent problems with a low efficacy and safety record [46].

Thus, it remains for future studies to determine which treatment strategies targeting the prevention of thromboembolic events and improved clinical outcomes in patients with AF may have a beneficial effect on long-term cognitive function. In any case, early detection of AF might prove useful in preventing cognitive impairment, particularly given the rapid progression of mild cognitive impairment (MCI) to full dementia [47] in AF patients.

### Symptom perception and symptom burden

Reflecting the heterogeneity of the AF disease condition itself, the AF symptom spectrum is highly variable, ranging from an asymptomatic (“silent”) condition to extremely troublesome symptoms which command immediate attention [48]. The symptom picture of AF encompasses both heart-specific symptoms (palpitations, heart flutter, skipping and jumping, chest discomfort) and more unspecific conditions (including fatigue, exhaustion, discomfort, shortness of breath, lightheadedness). Besides the variable nature of symptoms, they also vary in frequency, timing of onset, duration, and intermittent course [49].

The true proportion of silent AF in the general population is currently unknown, because AF screening even at repeated time points remains a random snapshot, unable to capture the wide range of paroxysmal AF episodes within an individual over time [50]. However, smartphone applications and other technological solutions (e.g., ECG Telecard) are currently under evaluation [51] in efforts to more accurately assess the true incidence AF despite the disease’s episodic nature and its propensity to remain asymptomatic. Silent and clinically apparent episodes may occur in parallel: in patients with paroxysmal AF, asymptomatic arrhythmia events occur even significantly more frequently than symptomatic arrhythmia events. A recent study of 50 patients with AF and implanted pacemakers [52] revealed that the majority (93%) of AF events were asymptomatic episodes, occurring 13 times as frequently as symptomatic (*p* < 0.001) events. Episodes lasting ≤ 5 min, male gender, lower heart rate, and a lower New York Heart Association (NYHA) class predisposed patients to silent atrial fibrillation. Unfortunately, the study did not consider psychological drivers of interoceptive sensing of internal bodily changes. Notably, the mortality risk associated with AF remains independent of the perceived symptom awareness and severity.

The “chaotic” symptom heterogeneity often precludes an adequate diagnostic strategy from first symptom awareness to a confirmed AF diagnosis and may result in a critically prolonged pre-diagnosis period, with a substantial proportion of patients experiencing unrecognized symptoms for more than 1 year, as shown in a qualitative investigation [48]. Here, two thirds of AF patients initially experienced complaints that were not readily perceptible and largely outside the conscious awareness: the “fleeting” nature of the symptoms made it difficult to decipher whether symptoms signaled a noticeable health problem [48]. A study of 150 AF patients (mean age 66.5 years) confirmed delayed treatment-seeking in the majority (70%, *n* = 105) of cases. Factors associated with delay included experiencing fatigue, dyspnea, intermittent symptoms, attributing symptoms to deconditioning, overwork, inadequate sleep, and perceiving symptoms as not very serious and amenable to self-management. Responses such as a wait-and-see approach, working through symptoms, reporting no fear of symptoms, or attempting to ignore symptoms were associated with delay. Unfortunately, this ambiguity is often mirrored in the physician’s daily routine: they likely dismiss the patient report as insignificant or attribute symptoms to benign conditions such as stress [53•].

However, once a firm AF diagnosis is established and patients receive professional attention, “*predictive coding*” [54, 55] takes place, through which adverse or positive illness expectations and the degree of self-mastery of the clinical course may sharpen or diminish symptom perception. A study with 99 AF patients (mean age 64.6 years) confirmed that acceptance of illness allowed patients to adapt to the disease and reduce symptom burden . Suffering from depression may lead to the opposite effect. In a prospective clinical study including 563 AF patients (319 patients with persistent AF and 244 with paroxysmal AF), a total of 35% patients suffered from depressed mood. Depression was associated with a substantial threefold increase in AF symptom burden (particularly in uneasiness, nausea, and shortness of breath). Of note, those patients who achieved measurable relief from depression after a follow-up period of 6 months also experienced reduced symptom burden (OR = 2.06; 95% confidence interval [CI]: 1.22 to 3.51) [56].

In caring for a patient with AF, current clinical guidelines advocate differentiating between treatment strategies based on either frequency or rhythm control. Therefore, the cardiologist must establish the pattern of arrhythmia and determine associated symptoms. However, given that psychological conditions (illness acceptance, depression) strongly influence AF symptom perception, a broader perspective than simply a focus on the electrophysiology alone is urgently needed.

### Psychological distress in patients with AF

In patients with confirmed AF, psychological distress (including anxiety, depression, and symptom preoccupation) is high, with estimates ranging from 25 to 50% [57]. Negative affect states due to arrhythmias are not limited to the AF event itself. Anxiety derived from fear of new episodes in patients with paroxysmal AF may in same cases become more limiting than the arrhythmia itself [58]. Nevertheless, in a cross-sectional comparison of patients enrolled in two large clinical trials, depression was significantly more common in patients with permanent AF than with paroxysmal AF [59]. In a sample of 170 patients with permanent AF [60], 35% of patients with high levels of anxiety and 20% with depressed mood were identified. Interestingly, both conditions were associated (among others) with “relations with medical staff.”

## Impact of psychosocial factors on the clinical course of AF

### Impact of negative affectivity on clinical outcomes

The relationship between AF and negative affectivity is bidirectional—AF symptom burden causes sustained psychological stress, but psychosocial discomfort may exacerbate the deterioration of the short- and long-term clinical course. Lange and Herrmann-Lingen [61] demonstrated that depressive symptoms were significant predictors of AF recurrence after cardioversion. A similar association was shown for anxiety and depression after circumferential pulmonary vein ablation [62]. A 1-year follow-up study after AF catheter ablation revealed an impact of type D personality (a phenotype of negative affectivity and social inhibition) on an adverse prognosis [63]. Analysis of long-term data from the Cardiovascular Research Network WAVE Study based on 25,570 adults with AF initiating warfarin treatment, among which 490 participants experienced ischemic stroke or intracranial hemorrhage (1.52 events per 100 person-years), revealed a strong association with anxiety, which was associated with a higher rate (adjusted HR 1.52) for these two endpoints. In contrast, neither isolated depression nor combined depression and anxiety were significantly associated with outcomes [60].

### Impact of sociodemographic factors on AF incidence and progression

Apart from mental psychosocial stress factors, socioeconomic and environmental social factors may also affect the onset and course of AF. By using US vital statistics for 55- to 89-year-old white (136,573 AF-related deaths) and black subjects (8288 AF-related deaths), Patton et al. [18] compared demographic AF-related mortality risk within the US stroke belt (SB), a disadvantaged region in the southeastern United States, against the national average AF-related mortality. They found an odds ratio of 1.19 in black and 1.09 in white subjects for AF-related mortality associated with SB birth. A subsequent study [64] confirmed these findings with data from 24,323 participants in the US Health and Retirement Study, but failed to identify an association with any specific childhood or adult cardiovascular risk factors, suggesting that there are as yet unidentified early life factors relevant to the development of AF.

In a study with 12,283 AF patients treated in primary care [65], low neighborhood socioeconomic status predicted higher relative risk of all-cause mortality (HR 1.49) compared to middle socioeconomic status neighborhoods. The authors confirmed these findings in the same data set over a 6-year follow-up period. They showed that high educational level was associated with *reduced* mortality in fully adjusted models: HR of 0.82 for college/university education versus primary school in men [66•]. Involuntary unemployment due to job loss has been associated with increased risk of cardiovascular events. Data from individuals enrolled in the REasons for Geographic And Racial Differences in Stroke Study [67] including 4273 participants without AF at baseline showed that unemployment was associated with incident AF (OR 1.54).

In summary, living in deprived neighborhoods, low levels of formal education, and involuntary unemployment are proxies for a low social class gradient, which contributes to an increased risk of AF onset or an adverse course of AF, thus raising important clinical and public health concerns. These data also show that AF is embedded as a disease condition in a societal context and is not an isolated medical problem per se.

### Quality of life as outcome variable in AF trials

The views of the physician and the patient on what they consider to be a “successful” therapeutic procedure may share only minimal agreement [68]. Therefore, it is now widely accepted that patient expectations and values regarding treatment should be part of patient-oriented health care, and patients’ preference of various treatment options should be integrated into clinical trials [69]. Among “patient-reported outcomes” in AF research, assessment of treatment satisfaction is one option, with questionnaires such as PACT-Q2 (Perception of Anticoagulant Treatment Questionnaire), which scores along two domains (convenience and anticoagulant treatment satisfaction). PACT-Q2 was applied in the multicenter ENSURE-AF study, a prospective randomized trial of anticoagulation for cardioversion in AF patients, to evaluate the impact of novel oral anticoagulant (NOAC) therapy on treatment satisfaction [70]. After 4 weeks, patients treated with the index medication were more satisfied than the control treatment group. Interestingly, differences in treatment satisfaction scores were significantly higher in patients who underwent non-transesophageal echocardiography (TOE)-guided cardioversion than in patients who underwent TOE-guided cardioversion.

Undoubtedly, “*health-related quality of life*” (HR-QoL) has received the most attention in recent years [71]. HR-QoL is a measure of how an individual's well-being may be affected by a disease condition over time. Regulatory authorities increasingly request data on HR-QoL for approval of new treatment modalities, and it is becoming an accepted criterion when evaluating health outcomes in AF treatment studies [72].

However, at least 34 different HR-QoL instruments are currently in use, suggesting a lack of consensus on an optimal approach [73]. Son et al. [74] describe generic, cardiac-specific, and AF-specific instruments which have recently been employed in randomized controlled trials (RCTs) in AF research. These data offer useful information on patient-reported outcomes. Wokhlu et al. [75], for example, investigated the relationship between AF ablation efficacy, HR-QoL, and AF-specific symptoms in 502 symptomatic AF ablation recipients and confirmed a sustained HR-QoL improvement at 2 years, interestingly across ablation outcomes, including recurrent AF. As an example of the benefit of applying tailored instruments rather than generic tools alone in order to more precisely capture the reality of life in AF, one study [76] prospectively assessed different aspects of short- and long-term HR-QoL after catheter ablation for AF, employing seven validated generic and tailored HR-QoL questionnaires. The investigators showed that QoL improved significantly 3 months after ablation in *all* patients (regardless of ablation success or AF type) and remained significantly improved after a median of > 4 years. These findings point to a potential major shortcoming of HR-QoL research attempting to evaluate the superiority of a treatment option under investigation: presumably, the impact of the underlying negative mental health condition—with AF-related anxiety in the first line [74]—may affect HR-QoL more than the primary treatment options under investigation.

With this in mind, it should be noted that in everyday routine, physicians may substantially underestimate the mental health burden of AF patients. We analyzed the degree of congruence between patients' and physicians' assessments of subjective health status in 334 patients with paroxysmal AF and found a significant discordance between physician and patient ratings as measured with the 12-item and 8-item Short Form Health Survey (SF-12 and SF-8). On average, physicians rated their patients' QoL significantly higher than did patients. Notably, discordance was substantial in depressed patients but also in patients reporting sleeping disorders and physical inactivity, pointing to deficits in communication and shared understanding [77].

## Gender aspects of coping with AF

Gender-related differences represent a pivotal factor resulting in distinct prevalence, clinical presentations, and therapy outcomes of AF. Although women have 30–50% lower incidence and prevalence of AF than men [1], women with AF often have atypical symptoms [78], worse QoL [79], and higher risk for adverse events (e.g., stroke and death) [80, 81].

While the exact mechanisms remain to be elucidated, evidence suggests the existence of sex-related differences in physiological, electrical, and structural characteristics of the atria, mainly in the pathological remodeling of cardiac tissue in atrial fibrillation. Animal models demonstrate differences in the electrophysiological properties between sexes [82]. Furthermore, research has shown a direct effect of sex hormones on ion channels and their expression, with implications for arrhythmogenesis and clinical outcome in AF [83]. Evidence also reveals gender-specific outcomes of antiarrhythmic drugs and catheter ablation, with more adverse effects in women than in men [84].

To date, only a few studies have assessed gender-related psychosocial aspects of AF. While prospective data from the Framingham Offspring Study cohort revealed that psychosocial stress was an independent risk factor for the development of AF in men but not women [16, 20], high psychosocial stress was associated with AF in women but not men in the Malmö Diet Cancer Study [10]. Data from the Women’s Health Study also showed a significant association between traumatic life events and AF [85], and positive affect was associated with reduced AF risk [12]. Similarly, in the PaTH AF cohort study (*N* = 953, mean age = 72 ± 10, 65% male), female sex was associated with higher AF symptom severity and greater symptoms of anxiety and depression [86]. Additionally, a study in patients with recurrent AF (*N* = 93) demonstrated that depression mediated the effect of gender on physical health, whereby the increased susceptibility of female AF patients to depressive symptoms might be a major contributing factor to their poorer physical health compared to their male counterparts [87]. Given the limited evidence thus far, future studies should focus on gender-related effects of psychosocial factors on AF in order to better understand the gender difference and facilitate prevention and intervention strategies.

## Role of psycho-neuro-biological conditions in AF

The growing awareness of an independent impact of stressful mental health conditions on the onset and adverse long-term course of AF calls for an anchoring of these findings in precise psycho-neuro-biological pathways. There is little doubt that adverse acute and sustained mental stress causes impairments in the autonomic nervous system and the endocrine and immune systems—notably neuromodulator conditions which also accompany the AF disease per se. For example, AF is characterized by systemic inflammation with elevated levels of inflammatory cytokines including tumor necrosis factor (TNF-alpha) and C-reactive protein (CRP) [88]—a phenomenon also convincingly shown for severe psychosocial stress of any kind. Thus, this coincidence gives room for the assumption that psychosocial stress—superimposed on mechanisms related to basic pathophysiological processes in AF—influences the onset and long-term course of AF. Although numerous investigations support this concept for cardiovascular disease and heart failure, research in the context of AF is still in its infancy, with most work so far focused on autonomic regulation [89].

Indeed, the heart is richly innervated by autonomic nerves. The ganglion cells of the autonomic nerves are located either outside (extrinsic) or inside (intrinsic) the heart. The extrinsic sympathetic nerves originate from the paravertebral ganglia, including the superior and middle cervical ganglion, the cervicothoracic (stellate) ganglion, and the thoracic ganglia. The stellate ganglion (major source of cardiac sympathetic innervation) connects with multiple intrathoracic nerves and structures. Like the stellate ganglion, the vagal nerves also have a complex structure containing mixed nerve types. Intrinsic cardiac nerves are mostly located in both atria and are therefore involved in arrhythmogenesis [90].

Animal studies support the importance of the autonomic nervous system in AF, showing that the stimulation of autonomic ganglia or the combined stimulation of sympathetic and parasympathetic nerves leads to spontaneous cell activity in the pulmonary veins [91, 92]. Several experimental investigations have found marked variations in autonomic tones before the onset of paroxysmal AF [93, 94] or atrial flutter [95]. AF onset is associated with simultaneous sympatho-vagal activation rather than with an increase in vagal or sympathetic drive alone [90].

Given that the autonomic nervous system contributes to AF substrate development and serves as a trigger of AF episodes, modulation of the autonomic nervous system is likely a promising strategy to protect the myocardium from pro-arrhythmic autonomic influences. Here, it is not the increase in parasympathetic tone alone, but the suppression of extreme fluctuations in both opposing autonomic nervous system components, which presumably is the basic mechanism that drives treatment results.

First evidence to support this conceptualization comes from a small prospective pre–post cohort study which evaluated the role of meditation (yoga), a noninvasive alternative medicine intervention, in patients with symptomatic paroxysmal AF. Yoga significantly reduced symptomatic and asymptomatic AF burden, improved anxiety and depression, and had a beneficial effect on heart rate and blood pressure [96, 97]. In several investigations, Stavrakis et al. revealed that low-level cervical vagus nerve stimulation (LLVNS) significantly suppressed AF inducibility and shortened AF duration [88]. They demonstrated (first in canines and later in patients referred for catheter ablation of paroxysmal AF) that AF inducibility was suppressed by LLVNS using a completely noninvasive approach by transcutaneous low-level stimulation of the tragus (LLTS), where the auricular branch of the vagus nerve is easily accessible. They recently confirmed these findings in an RCT [98]. After 6 months of therapy, the AF burden was 85% lower in patients who underwent stimulation versus controls. Of note, an anti-inflammatory effect of LLTS was also shown in these patients. Recently, Lampert et al. [99••] performed a 12-month electronic diary-based study with 95 patients suffering from paroxysmal AF. This investigation showed that negative emotions in daily life increased the likelihood of an AF episode by two- to fivefold. Notably, prescription of beta-receptor blockers significantly counteracted the triggering effects of anger or stress.

## Conclusions

This review provides evidence of the involvement of psychological conditions in the etiology of AF onset and prognostic impact on its clinical course. It shows that severe job strain, long working hours, and non-occupational perceived stress, independent of major somatic risk factors, predict AF onset in large-scale longitudinal population-based studies. Likewise, psycho-traumata and depressive symptoms demonstrate an influence, which is however stronger in prospective clinical studies with established AF disease, especially in female patients. Promising yet preliminary data show that underprivileged populations are at greater risk of developing AF. A deeper understanding of negative affect comorbidities in the evolving progression of the disease is likely to aid in optimizing treatment and self-regulation of AF patients. There is a paucity of evidence-based interventions for behavioral problems in AF patients. However, non-pharmacological treatment options aiming at increasing coping skills and suppression of interoceptive symptom cues may contribute to a balanced modulation of the autonomic nervous system, which apparently plays a crucial role in AF substrate development and triggering of AF episodes. In summary, an integrated care approach, with thorough consideration of psychological aspects of the AF patient in clinical routine, is urgently needed and should not be viewed as a dispensable adjunct.
